# An unusual unifocal presentation of Castleman’s disease in a young woman with a detailed description of sonographic findings to reduce diagnostic uncertainty: a case report

**DOI:** 10.1186/1756-0500-6-97

**Published:** 2013-03-15

**Authors:** Norbert Wagner, Zerrin Maden

**Affiliations:** 1 Department of Obstetrics and Gynecology, Marienhospital Essen, Hospitalstrasse 24, Essen 45329, Germany; 2 Department of Obstetrics and Gynecology, University Hospital Frankfurt, Theodor Stern Kai 7, Frankfurt 60590, Germany

**Keywords:** Castleman’s disease, Giant lymph node hyperplasia, Ultrasonography, Core needle biopsy

## Abstract

**Background:**

Castleman’s disease is a rare lymphoproliferative disorder. It typically presents as mediastinal masses and causes a wide range of clinical symptoms. Histologically, Castleman’s disease is classified as either a hyalinic vascular or plasma cell variant. The prognosis mainly depends on the histological type and broadly varies. We herein report our sonographic findings in a patient with Castleman’s disease, including gray-scale ultrasonography, color Doppler ultrasonography, and sonoelastography ultrasonography, which have not been previously reported in the literature. These findings allowed for a preoperative diagnosis and avoidance of overly aggressive therapy.

**Case presentation:**

A 28-year-old European female patient with unicentric Castleman’s disease of hyalinic vascular type (HV) restricted to the axilla was referred to us because of a 4-month history of a painless, solitary mass located in the left axilla. The patient’s medical history was unremarkable.

**Conclusion:**

Castleman’s disease is a pathologic entity of unknown etiology and pathogenesis. In this case report of unicentric HV-type CD, we demonstrate that typical sonographic findings can lead to a preoperative diagnosis of Castleman’s disease. Core needle biopsy usually allows for a final diagnosis and helps to avoid unnecessary operations and overtreatment.

## Background

Castleman’s disease (CD), or giant lymph node hyperplasia, is a rare lymphoproliferative disorder that typically presents as mediastinal masses. It was first described by Castleman and colleagues as a localized mass of mediastinal lymphoid follicles in 1954. Two years later, it was defined as a pathologic entity of unknown etiology and pathogenesis [1,2].

Clinically, CD may be localized with no major symptoms and present as a solitary mass or swelling, or it may be a generalized, symptomatic disease with fever, weight loss, anemia, hepatosplenomegaly, and generalized lymphadenopathy.

Histologically, the disease is also classified into two separate subtypes: the hyalinic vascular (HV) variant (80%–90% of cases) and the plasma cell (PC) variant (10%–20% of cases). Intermediate and mixed types have also been reported.

The prognosis mainly depends on the histological type and shows a broad variety. Treatment can range from curative surgery for the solitary form to the use of steroids, monoclonal antibodies, chemotherapy, and radiotherapy for the multicentric type [3].

We herein report on a 28-year-old female patient with unicentric CD restricted to the axilla. We describe the findings and imaging features of gray-scale ultrasonography (US), color Doppler US, sonoelastography US, and contrast-enhanced dynamic computed tomography (CT). A pathway to a preoperative diagnosis, management of the disease, and the clinical course are presented. A review of the literature and differential diagnoses are also presented.

## Case presentation

A 28-year-old European female patient was referred to us because of a 4-month history of a painless, solitary mass located in the left axilla. She had no accompanying complaints, history of fatigue, night sweats, or weight loss. Her medical history was unremarkable.

On routine physical examination, a solitary enlarged lymph node was detected in the left axilla in the absence of any breast pathology. No generalized lymphadenopathy or other organomegaly was noted. Peripheral blood counts and the erythrocyte sedimentation rate were within normal limits. Interestingly, the levels of lymphoproliferative markers such as serum soluble IL-2R, beta 2-microglobulin, and immunoglobulins were also normal; however, the C-reactive protein level was slightly increased.

The lymph node in the left axilla measured 4 cm and was mobile, nontender, and soft in consistency. US examination of the breast and axilla was performed with an iU22 (Philips Healthcare, Bothell, WA) and ProSound 7 (Aloka, Hitachi, Zug, Switzerland) using a 12-MHz linear array transducer.

**Figure 1:**
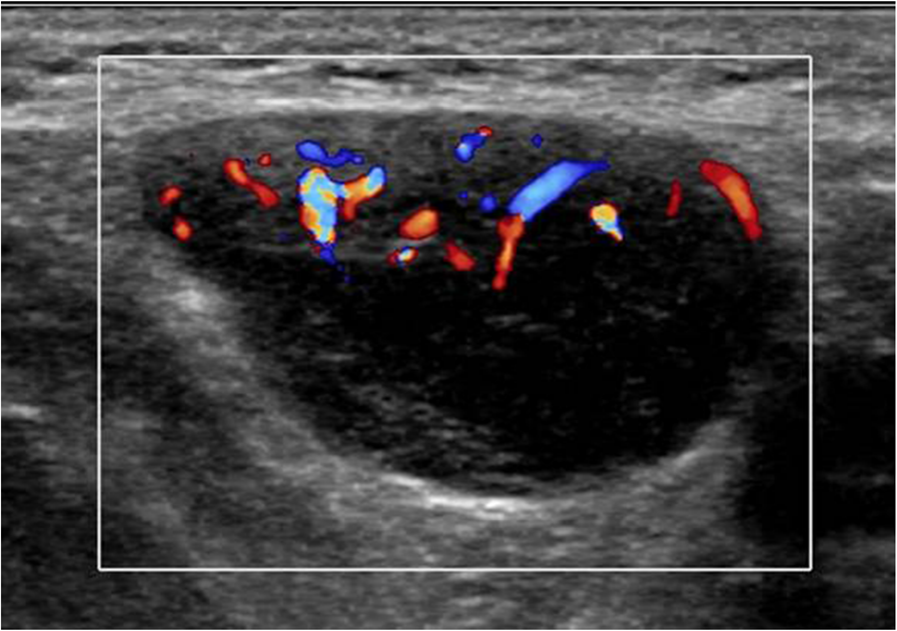
Color Doppler sonogram shows peripheral vascular flow within an ovoid hypoechoic axillary lymph node.

High-frequency, high-resolution gray-scale US revealed a well-defined, uniformly hypoechoic, ovoid axillary mass, 38 × 17 × 28 mm in size. The longitudinal diameter was greater than the transverse diameter with a longitudinal to transverse axis ratio of more than 2. A hyperechoic fatty hilum could not be detected and was totally replaced by cortical thickening. Although soft in consistency, the lesion could only be slightly deformed by compression with the transducer. Color Doppler flow was performed with optimized color Doppler parameters set at a low wall filter (80–100 Hz) and low velocity scale (pulse repetition frequency, 1000 Hz). Color gain was adjusted dynamically to maximize depiction of blood vessels while avoiding artifactual color noise. Bizarre and multifocal peripheral flow was detected, whereas central or central perihilar flow was not revealed (Figure 1). A three-dimensional and multislice imaging scan with the capability of reproducing high-resolution images confirmed these B-mode findings, but could not provide additional important information.

Spectral Doppler analysis along the periphery of the node showed both arterial and venous pulse wave patterns. The blood flow profile of the arteries indicated a broad range in the resistance index, pulsatility index, and peak systolic velocities varying from low to high pulsatility. Thus, no further information could be drawn on these indices.

Sonoelastography US confirmed the clinical examination findings: the lesion was characterized by soft tissue with some less elastic regions of higher stiffness.

**Figure 2:**
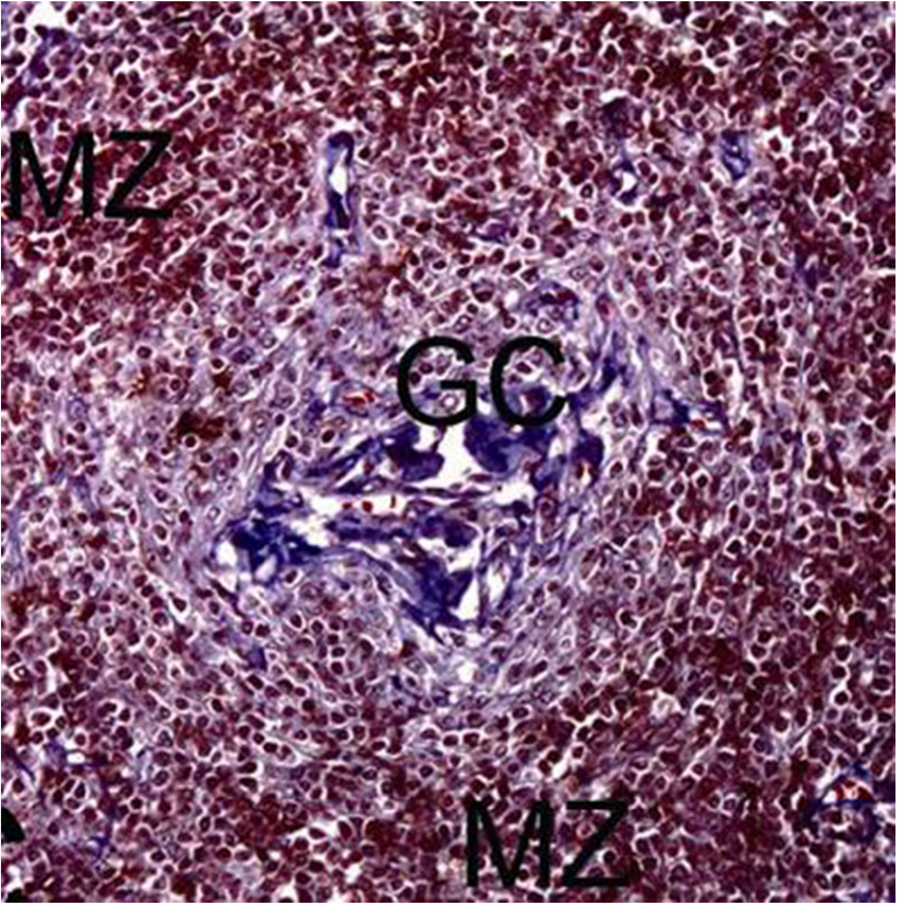
**Histopathologic specimen shows a continuum of abnormal germinal centers (GC) with prominent CD 23-positive follicular dendritic cells (dense black brown staining) and subtle vascular proliferation (original magnification, ×200).** MZ, mantle zone.

An US-guided fine needle biopsy with multiple passages of the needle tip through the nodal cortex was made to sample as much of the nodal cortex as possible. Fine needle aspiration cytology (FNAC) only revealed a mixed population of small and large lymphoid cells. In particular, prominent vascularity with hyalinized capillaries was not detected. The FNAC results were subsequently reported as “negative for malignant cells,” and histopathologic examination of the lymph node was advised. Therefore, US-guided core needle biopsy using a 14-G automated gun was performed, and a diagnosis of HV-type CD was confirmed: microscopic examination revealed many variably sized hyperplastic follicles, progressive vascular proliferation, and hyalinization (Figure 2).

**Figure 3:**
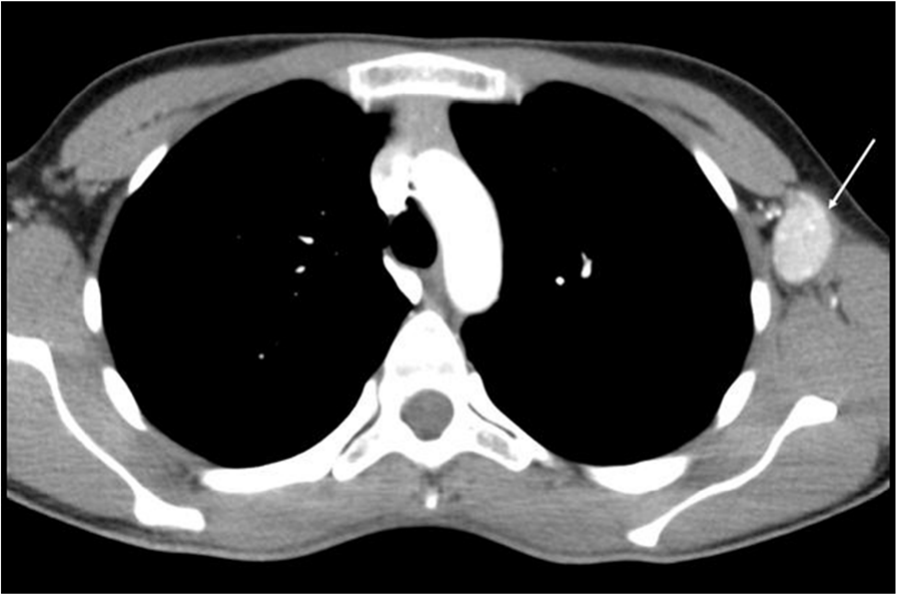
CT scan shows enlarged lymph node (arrow) in the left axilla.

**Figure 4:**
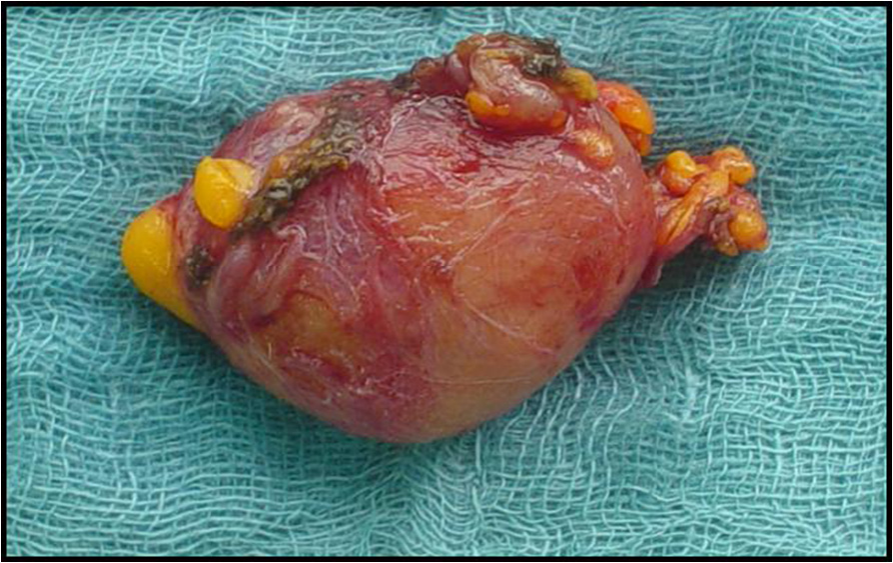
Macroscopic aspect of affected lymph node measuring 4.5 cm.

A multislice CT scan of the head, thorax, and abdomen was subsequently performed and allowed for the exclusion of multicentric type CD. The lymph node in the left axilla on CT was described as a well-circumscribed, homogeneous mass lesion with moderate to intense enhancement and rapid washout (Figure 3). The patient underwent open biopsy by a surgical gynecologist, and the enlarged axillary lymph node was completely excised (Figure 4). The postoperative course was uneventful, clinical follow-ups were unremarkable, and there has been no evidence of recurrence.

## Discussion

CD is a rare, benign lymphoproliferative disorder of unknown etiology. The two main hypotheses for its development are an abnormal immune response and viral infection. Human herpes virus 8 and interleukin 6 are regarded to be linked to the pathogenesis [4]. Microscopically, as stated above, two histological subtypes are known: the HV type and PC type. Depending on the clinical presentation, CD can also be divided into a localized and multicentric type. About 90% of the localized type belongs to the HV subgroup, as seen in our patient, and almost all of the multicentric type is histologically the PC subtype.

CD can develop anywhere that lymphoid tissue is found, most commonly in the mediastinum (60%), but also in the abdomen, neck, lung, and retroperitoneum. Less than 4% of cases present as a lymph nodal mass in the axilla [5]. Patients with the HV type are usually asymptomatic, as our patient was, whereas patients with the PC type typically present with a broad variety of symptoms such as fever, weight loss, generalized lymphadenopathy, night sweats, and hepatosplenomegaly. As also demonstrated in our case, localized CD is normally cured after excision of the tumor with an excellent prognosis and 5-year survival of approximately 100%. On the other hand, successful treatment of the multicentric type often requires multimodal management including radiotherapy, chemotherapy, and surgery. The prognosis is generally less favorable [4].

In most cases of CD, as in our patient, US shows a hypoechoic, well-circumscribed homogeneous mass lesion [6]. In all reported cases, the longitudinal to transverse axis ratio of involved lymph nodes was more than 2 and was significantly higher in benign than in malignant lymph nodes [7]. Color Doppler findings are characteristic for the diagnosis of CD: prominent peripheral vascular proliferation in the node is not seen in healthy or reactive lymph nodes and is absent from lymph nodes affected by malignancy. Reactive lymph nodes are more likely to preserve a normal vascularity pattern with central hilar vessels, whereas lymph nodes in patients with CD show bizarre new blood vessels in the periphery due to neovascularization, as in our patient [8]. Histologically, polymorphous lymphoreticular infiltrates containing numerous capillaries are seen at the periphery of the lymph node. Malignant lymph nodes typically present a mixed vascular distribution including both central and peripheral flow.

These US and Doppler findings, although nonspecific, seem to be characteristic for the diagnosis of this uncommon disease entity and may help to differentiate this benign process from reactive lymph nodes and nodal metastases. These US findings must be proven to be efficacious, and larger studies of patients with CD are required to determine the role of US and sonoelastography in this group. Whether the distribution of nodal vascularity and Doppler flow characteristics can help to achieve a better understanding of CD must be assessed.

As in our case, CD is difficult to diagnose based on aspirate material. FNAC as the initial investigation method may be misleading because no specific cytomorphological criteria for a definitive diagnosis have been described, nor are there any cytomorphological features pathognomonic for the disorder [9].

Another technique for preoperative axillary node diagnosis is US-guided core biopsy. Although more expensive and invasive, resulting in a higher complication rate, core biopsy has the advantage of sampling the nodal tissue more extensively than using FNAC. Using core needle biopsy and excision biopsy, all cases reported in the literature were diagnosed as CD [9]. As in our patient, core needle biopsy was superior to FNAC and gave the correct definitive diagnosis.

The differential diagnoses of an axillary mass include metastases, lymphoid neoplasms such as Hodgkin’s lymphoma and non-Hodgkin lypmphoma, and a number of reactive, inflammatory, and nonmalignant conditions such as rheumatoid arthritis, Wiskott-Aldrich syndrome, tuberculosis, sarcoidosis, syphilis, and other disorders of immune regulation in patients with acquired immune deficiency syndrome and Kaposi’s sarcoma. Because of its variable clinical presentation, CD should be considered as a differential diagnosis of any enlarged lymph node.

## Conclusion

In conclusion, although it is probably not possible to render a definitive diagnosis of CD based on US findings, the presence of a hypoechoic lymph node with many prominent peripheral vessels on Doppler sonogram should at least raise the diagnostic possibility. This case report highlights the difficulty in diagnosing unicentric CD in FNA samples. Core needle biopsy, which usually achieves the final diagnosis, should be given preference. As shown in our case report, unicentric CD should be a differential diagnosis of an enlarged lymph node, especially in asymptomatic and young patients. Surgical removal of the affected lymph node is curative in localized HV-type CD. Confirmation of CD should be based upon the combination of clinical, sonographic, CT, and histopathological findings.

## Consent

Written informed consent was obtained from the patient for publication of this manuscript and accompanying images. A copy of the written consent is available for review by the Editor-in-Chief of this journal.

In this original case report, we first describe the findings and imaging features of Castleman’s disease based on gray-scale ultrasonography (US), color Doppler US, sonoelastography US, and contrast-enhanced dynamic computed tomography. The description of the sonographic findings in this unique case of Castleman’s disease of the axilla will certainly advance our understanding of this illness.

## Competing interests

The authors declare that they have no competing interests.

## Authors’ contributions

NW and ZM performed the clinical work, data collection, and data analysis. Both authors read and approved the final manuscript.
